# Factors associated with self-reported medical errors among undergraduate health science students in southern Ethiopia

**DOI:** 10.3389/fmed.2024.1354270

**Published:** 2024-06-13

**Authors:** Kusse Koirita Toitole, Fekade Tesfaye Danaso, Saron Assefa Alto, Tofik Mohammed, Sisay Dejene, Wanzahun Godana Boynito

**Affiliations:** ^1^Department of Internal Medicine, Arba Minch General Hospital, Arba Minch, Ethiopia; ^2^Nguenyyiel Refugee Health Project, Medecins Sans Frontieres (Doctors Without Borders), Gambella, Ethiopia; ^3^Department of Forensic Medicine and Toxicology (FMT), St. Paul's Hospital Millennium Medical College, Addis Ababa, Ethiopia; ^4^Department of Internal Medicine, College of Medicine and Health Sciences, Arba Minch University, Arba Minch, Ethiopia; ^5^School of Public Health, College of Medicine and Health Sciences, Hawassa University, Hawassa, Ethiopia; ^6^School of Public Health, College of Medicine and Health Sciences, Arba Minch University, Arba Minch, Ethiopia; ^7^Department of Public Health and Primary Care, Faculty of Medicine and Health Sciences, Ghent University, Ghent, Belgium

**Keywords:** patient safety, knowledge, attitude, practice, health science students, healthcare workers, medical errors, Ethiopia

## Abstract

**Introduction:**

Medical errors are not uncommon, but they are seldom reported. Patient safety practices are among the key areas for service improvement. This study aimed to assess factors associated with self-reported medical errors among undergraduate health science students in southern Ethiopia.

**Methods:**

A facility-based cross-sectional study was conducted among health science students of Arba Minch University in 2018. The sample size was calculated using a single population proportion formula. A total of 287 medical students in their fourth year and above, nursing and midwifery students in their second year and above, and other health science students in their third year and above were included.

**Results:**

The majority (82.1, 95%CI: 77.63–86.67) of the study participants had a ‘good’ knowledge score on patient safety. Approximately 62.5% (95%CI: 56.8–68.2) of the participants had a ‘favorable’ patient safety attitude. Only 38.6% (95%CI: 32.8–44.3) of the study participants had ‘good’ patient safety practices. At adjusted analysis, the practical attachment unit, having ever managed a patient independently, having ever witnessed harm to patients by colleagues or other healthcare workers, and having ever witnessed harm to a close friend or family member were statistically significantly associated with self-reported medical errors. Participants who were doing their practical clinical attachment in the surgical and obstetric units were three times more likely to self-report medical errors as compared to those practicing in the pediatrics, internal medicine, and other units (AOR = 2.72, 95%CI: 1.16–6.39.97). Students who had never managed a patient independently were less likely to self-report medical error (AOR = 0.24, 95%CI: 0.08–0.72). The odds of self-reporting medical errors were less among among participants who had not ever witnessed harm to patients by colleagues or other healthcare workers (AOR = 0.12, 95%CI: 0.05–0.29) and participants who had not ever witnessed harm to a close friend or family member (AOR = 0.36, 95%CI: 0.16–0.80).

**Conclusion:**

One in five of the participants reported having harmed patients while practicing. Most of the students had good patient safety knowledge, while approximately two-thirds of the participants had a favorable attitude toward patient safety. Only 38.6% of the study participants had good patient safety practices. Having worked in surgical and obstetrics units, having managed a patient independently, and having witnessed harm to a patient were associated with self-reporting of medical errors.

## 1 Introduction

Due to advancements in the knowledge of diseases and technological innovations, healthcare outcomes have significantly improved over the past two decades, resulting in increased life expectancy (1). Delivering safer care in complex and fast-moving environments has become a prominent challenge in the current healthcare systems. Adverse events do occur to patients during routine healthcare deliveries, and sometimes unintentional but serious life-threatening and lethal harm can happen to patients. One of the focuses of modern medicine is to minimize risks by advancing the culture of patient safety. The World Health Organization (WHO) defines patient safety as “the reduction of risk of unnecessary harm associated with healthcare to an acceptable minimum” (2–4).

Developed countries have better patient safety information systems, enabling the assessment of the magnitude of healthcare-related errors and facilitating improvement strategies by involving healthcare providers, families, and patients (5). In one study in the United States, there were an estimated 161,655 and 170,201 medical errors in the year 2008 and 2009, respectively (6), and another similar study in the United States estimated that medical errors in 2008 resulted in a $19.5 billion cost to the US economy (7). The authors of this study estimated that the economic impacts due to deaths resulting from medical errors may reach nearly $1 trillion annually when quality-adjusted life years (QALYs) are calculated for those who die.

The measurement of the magnitude of harm resulting from unsafe healthcare in developing countries is challenging because of the limited resources available, and most measurement activities rely on patient charts and other weak documentation systems (8). An evaluation of 26 hospitals across the WHO African and Eastern Mediterranean regions found that half a million people were victims of unsafe healthcare, with over 10,000 estimated deaths (4). One study at a pediatric ward of a university hospital in Ethiopia found that there were 9.2 adverse drug events per 100 hospital admissions, and from these, approximately 35% could have been prevented (9).

Studies have shown that patient safety is a relatively new focus in the Ethiopian health system, and there is little known about its scope (10, 11). A study that assessed pharmacy students’ attitudes toward patient safety in a university hospital in northern Ethiopia found that only half of the students agreed that pharmacists should discuss and report errors to an affected patient and their caregiver even if there was no harm to the patient (12).

With the growing recognition of the harms caused by healthcare, there has been an increased focus on the importance of teaching patient safety in medical education (1, 13). A report from the Institute of Medicine emphasized that incorporating patient safety education into clinical training programs is a key mechanism for improving patient safety (14).

This study aimed to assess factors associated with self-reported medical errors among undergraduate health science students of Arba Minch University.

## 2 Materials and methods

### 2.1 Study area and period

The study was conducted among undergraduate health science students at the College of Medicine and Health Sciences (CMHS) of Arba Minch University (AMU). Arba Minch town is located 505 km to the south of Addis Ababa, the nation’s capital. At the time this study was conducted, the town had one general hospital and two government (public) health centers. The CMHS initially started training in three departments in 2008 as the Faculty of Health Sciences. It was then upgraded to college level to train different health professionals, including medical doctors. The college started training medical doctors in the year 2009, and by the time this study was conducted, CMHS had 1749 regular students. After the 2nd, 3rd, 4th, or 5th year of their education, depending on the specific department they are studying, students from the college go to various hospitals and health centers located outside Arba Minch town to complete their practical clinical attachments.

The study period of this study was from 15 September to 15 October 2018.

### 2.2 Study design

The study design was an institution-based cross-sectional study.

### 2.3 Source population

The source population included all undergraduate students at the College of Medicine and Health Sciences (CMHS) of Arba Minch University.

### 2.4 Study population

The study population comprised randomly selected undergraduate students.

### 2.5 Study unit

The student unit comprised individual students.

### 2.6 Inclusion and exclusion criteria

#### 2.6.1 Inclusion criteria

Medical students in their fourth year and above, nursing and midwifery students in their second year and above, and other health science students in their third year and above were included in this study.

#### 2.6.2 Exclusion criteria

Students who were ill or absent at the time of data collection were excluded from this study.

### 2.7 Sample size determination

The total sample size was determined using a single population proportion formula with a 95% confidence interval and marginal error of 5%, considering the proportion of students who reported medical errors themselves to be 50%. Using these parameters, the estimated sample size was 384, and as the source population was less than 10,000, a finite population correction formula was used to yield a sample size of 261. Considering a 10% non-response rate, the final sample size was 287.

### 2.8 Sampling technique and procedure

A stratified sampling technique was used by dividing students into different departments, and the total sample size was proportionally allocated to each department. Then, a simple random sampling technique was employed to select study participants from each stratum using their respective lists.

### 2.9 Data collection instrument, data collectors, data collection, and data quality control

The data collection tool was adapted from the WHO patient safety curriculum guide and patient safety manuals, and adjustments were made to fit into the local study context (1, 4). Pre-tested structured self-administered questionnaires were used to collect data from a selected sample of students (See Supplementary Material). The questionnaire developed by the research team has four parts: socio-demographic characteristics, knowledge of patient safety, attitude toward patient safety, and practice of patient safety. The questionnaire consisted of 18 socio-demographic and clinical experience questions, 10 knowledge questions, 10 attitude questions, and 10 practice questions. It was pre-tested on 5% of the sample size among students from Hawassa University Comprehensive Specialized Hospital before the actual data collection. Training of the data collectors was conducted to ensure a common understanding of the data collection tools and to improve the quality of the data. The research team facilitated and supervised the data collection process.

### 2.10 Data analysis procedures

After the data were collected, the filled questionnaires were checked and organized by the research team members. The data were entered into EpiInfo version 3.5.1 and then exported into STATA version 15 for analysis. Descriptive statistics including frequencies and mean were used to provide a general description of the data.

For the knowledge questions, participants who correctly answered more than 80% of the knowledge questions were considered to have ‘good’ knowledge of patient safety, and those who answered less than 80% were considered to have ‘poor’ knowledge. For the attitude questions, participants who responded ‘strongly agree’ or ‘agree’ to the attitude questions were considered to have a ‘favorable’ attitude toward patient safety and those who responded ‘neutral,’ ‘disagree,’ or ‘strongly disagree’ were considered to have an ‘unfavorable’ attitude. Regarding practice questions, participants who self-reported their practices as competent, proficient, and expert were considered to have ‘good’ patient safety practice, and those who responded not competent or somewhat competent were considered to have ‘poor’ practice.

Bivariable and multivariable logistic regression analyses were used to identify factors associated with self-reported medical errors. Variables that were deemed to have theoretical relevance were included in the final model. The 95% confidence interval and odds ratio were used to determine significance.

### 2.11 Ethical consideration

Before the data collection, ethical clearance was obtained from the Institutional Research Ethics Review Committee of the College of Medicine and Health Sciences of Arba Minch University. Additionally, a support letter was obtained from the college to facilitate cooperation from the clinical attachment sites (hospitals and health centers).

Consent was obtained from each participant before the data collection. The respondent’s privacy and confidentiality of the information were assured throughout the study procedure, and participants had all the right not to be involved in the study or not to answer any of the questions. The data were handled with care, and access to the collected data was restricted only to the research team members.

## 3 Results

### 3.1 Socio-demographic characteristics and clinical experience-related factors

A total of 280 study participants were involved in the study, which is a response rate of 97.5%. Among the participants, 193 (53.6%) were men, and the majority of the students were in the age range of 21 to 24 years. The mean age of the participants was 22.6 years with a standard deviation of 1.8 years. The youngest age was 19, and the oldest was 28 (Table 1).

**Table 1:** Socio-demographic characteristics of undergraduate health science students of Arba Minch University.

Factors	Category	Frequency (*n*)	Percent (%)
Age	<=20	27	9.6
	21–24	208	74.3
	> = 25	45	16.1
Sex	Male	193	68.9
	Female	87	31.1
Religion	Orthodox Christian	150	53.6
	Muslim	43	15.4
	Protestant	81	28.9
	Other	6	2
Marital status	Unmarried	11	3.9
	Married	267	95.4
	Other	2	0.7
Family residence	Urban	210	75
	Rural	70	25

One-third of the students [90 (32.1%)] were studying medicine, 56 students (20.0%) were in nursing programs, and 46 students (16.4%) were in health officer programs. With regard to their respective clinical attachment sites, 175 participants (62.5%) had their attachments at hospitals, whereas 105 participants (37.5%) had their attachments at health centers (Table 2).

**Table 2:** Study field, clinical attachments, and attachment duration among undergraduate health science students of Arba Minch University.

Variables	Category	Frequency (*n*)	Percent (%)
Field of study	Medicine	90	32.1
	Health Officer	46	16.4
	Nurse and Midwifery	78	27.9
	Medical Laboratory	30	10.7
	Anesthesia	36	12.9
Year of study	2nd year–3rd year	109	38.9
	4th year–5th year	114	40.7
	6th year	57	20.4
Place of attachment	Hospital	175	62.5
	Health Center	105	37.5
Duration of Stay in Current Hospital/Health Center	<= 6 months	200	71.4
	7–12 months	38	13.6
	13–24 months	16	5.7
	> = 25 months	26	9.3
Duration of practical Attachment	<6 month	66	23.6
	month–12 month	95	33.9
	13–24 months	54	19.3
	> = 25 months	65	23.2
Current attachment unit	Pedi/Medi	62	22.1
	Surg/Obs	129	46.1
	Others	89	31.8

In total, 46 (16.4%) of the study participants had no clue about the term ‘Patient Safety’, whereas 119 (42.9%) reported being encouraged by their teachers or supervisors to report medical errors. Nearly two-thirds of the participants [198 (70.7%)] had experience in managing patients independently, while 136 (48.6%) did not take formal training on patient safety; one in five (18.2%) of the study participants self-reported having caused harm to patients while practicing. More than one-third of the study participants [103(36.8%)] reported having witnessed harm to patients by health workers, and 84 (30%) reported having witnessed harm to a close friend or family member (Table 3).

**Table 3:** Information regarding patient safety characteristics of undergraduate health science students of Arba Minch University.

Factors	Category	Frequency (*n*)	Percent (%)
Ever heard about patient safety	Yes	234	83.6
	No	46	16.4
Training on patient safety	Yes	144	51.4
	No	136	48.6
Ever managed patients independently	Yes	198	70.7
	No	82	29.3
Supervisors encourage reporting of medical errors	Yes	161	57.5
	No	119	42.5
Ever done harm to patients	Yes	51	18.2
	No	229	81.8
Ever witnessed harm to patients by colleagues	Yes	103	36.8
	No	177	63.2
Ever witnessed harm to a close friend or family member	Yes	84	30
	No	196	70

### 3.2 Knowledge, attitude, and practice levels toward patient safety

The majority [230 (82.1%), 95%CI: 77.6–86.7)] of the study participants had a ‘good’ knowledge score on patient safety (correctly answered 80% or above of the knowledge questions). Approximately two-thirds (62.5, 95%CI: 56.8–68.2) of the participants had a ‘favorable’ attitude about patient safety (responded ‘strongly agree’ or ‘agree’ to the attitude questions). Only 38.6% (95%CI: 32.8–44.3) of the study participants had ‘good’ patient safety practice (responded as ‘competent,’ ‘proficient,’ or ‘expert’ to the practice questions).

### 3.3 Factors associated with self-reported medical errors

In total, 51 (18.2%) of the study participants responded, ‘yes’ to the question ‘Have you ever done harm to patients while you are practicing?’

In the bivariable analysis, factors such as having ever managed patients independently, witnessing harm to patients by healthcare workers, and witnessing harm to a close friend or family member were found to be statistically significantly associated with self-reported medical errors (Table 4). Participants who had not managed a patient independently were less likely to cause harm to a patient (COR = 0.33, 95%CI: 0.14–0.76). The odds of self-reported medical errors were lower among students who had not ever witnessed harm to a patient by a colleague or other healthcare workers (COR = 0.12, 95%CI: 0.11–0.24) and among participants who had not ever witnessed harm to a close friend or family member (COR = 0.19, 95%CI: 0.10–0.37).

In the multivariable or adjusted analysis, factors such as the practical attachment unit (department), having ever managed a patient independently, having ever witnessed harm to patients by colleagues or other healthcare workers, and having ever witnessed harm to a close friend or family member were found to be statistically significantly associated with self-reported medical errors. Participants who were doing their practical clinical attachment in the surgical and obstetric units were three times more likely to self-report medical errors as compared to those practicing in other departments (AOR = 2.72, 95%CI: 1.16–6.39). Students who had not ever managed a patient independently were 76% less likely to self-report medical errors (AOR = 0.24, 95%CI: 0.08–0.72) compared to their counterparts. The odds of self-reporting medical errors were less among participants who had not ever witnessed harm to patients by colleagues or other healthcare workers (AOR = 0.12, 95%CI: 0.05–0.29) and among participants who had not ever witnessed harm to a close friend or family member (AOR = 0.36, 95%CI: 0.16–0.80).

In the adjusted analysis, there was no statistically significant association between self-reported medical errors and patient safety knowledge, attitude, or practice level.

**Table 4:** Factors associated with self-reported medical errors among undergraduate health science students at Arba Minch University (*n* = 280).

Variables	Categories	Ever done harm to patients		COR (95%CI)	AOR (95%CI)
		Yes	No		
Current attachment unit	Pedi/Medi/Others	24	127	1	1
	Surg/Obs	27	102	1.40 (0.76–2.57)	2.72 (1.16–6.39) *
Ever managed patients independently	Yes	44	154	1	1
	No	7	75	0.33 (0.14–0.76) *	0.24 (0.08–0.72) *
Ever witnessed harm to patients by colleagues	Yes	39	64	1	1
	No	12	165	0.12 (0.11–0.24) *	0.12 (0.05–0.29) *
Ever witnessed harm to a close friend or family member	Yes	31	53	1	1
	No	20	176	0.19 (0.10–0.37) *	0.36 (0.16–0.80) *
Knowledge level	Good	40	190	0.75 (0.35–1.58)	0.48 (0.17–1.35)
	Poor	11	39	1	1
Attitude level	Favorable	32	86	1.01 (0.54–1.91)	0.99 (0.43–2.30)
	Unfavorable	19	143	1	1
Practice level	Good	20	88	1.03 (0.55–1.93)	1.36 (0.63–2.95)
	Poor	31	141	1	1

*p-value less than 0.05; CI, confidence interval; COR, crude odds ratio; AOR, adjusted odds ratio.

## 4 Discussion

A considerable number of participants reported having caused harm to patients while practicing. Most students had good patient safety knowledge, while approximately two-thirds of the participants had a favorable attitude toward patient safety. Over one-third of the study participants had good patient safety practices. Practical attachment unit (surgery and obstetrics units), having ever managed a patient independently, having ever witnessed harm to patients by colleagues or other healthcare workers, and having ever witnessed harm to a close friend or family member were found to be statistically significantly associated with self-reported medical errors.

In this study, 18.2% of the participants reported having caused harm to patients while practicing. This finding was lower than studies conducted among medical interns, residents, and physicians in Korea and Poland where participants who self-reported medical errors were 59% and 63.7%, respectively, (15, 16). One study conducted among nurses in public health hospitals in southern Ethiopia found that 71% of the nurses admitted to making medication administration errors, while a similar study among nurses at a referral hospital in northern Ethiopia found the rate of medication errors to be 29.1% (17, 18). The discrepancies can be explained by the fact that medical interns, residents, nurses, and physicians are more involved in patient management as compared to health science students in the initial stages of their practical attachment. In addition, the patient safety culture and awareness level in the different settings may impact the attitude and practice toward self-reporting medical errors.

In this study, 82.1% of the participants had good knowledge about patient safety. This finding is higher than a result from a study in a health center in northern Ethiopia, which found that 47% of the participants had inadequate knowledge about reporting adverse drug reactions (19). Another study among medicine, nursing, and midwifery students in Iran found that 50% of the students had demonstrated good knowledge regarding patient safety (20). A separate study among community pharmacists in Lebanon found that the majority of the participants had good knowledge of the concept and purpose of pharmacovigilance and drug reactions (21). The discrepancies between the findings of this study and the other studies in Ethiopia, Iran, and Lebanon could be due to the differences in the type of questions regarding patient safety, the level of understanding of the students and health professionals in different settings, and the differences in the departments of study.

The findings from this study revealed that approximately two-thirds (62.5%) of the participants had a favorable attitude toward patient safety. This finding is comparable to a finding from a study among community pharmacists in Lebanon, which found that the majority of the respondents had a positive attitude toward their responsibility and duty in reporting adverse drug reactions (21). The results of this study are lower than that of another northern Ethiopian study, which found an 84.3% positive attitude level of patient safety among pharmacy students (12). The differences in the findings between this study and the other studies could be explained by differences in the study setting and the professional level of the study participants.

The overall good practice level of the participants in this study was 38.6%. This finding is comparable to results from a study among community pharmacists in Lebanon, which found a lack of practice on pharmacovigilance and medication safety among the study participants (21). Another study among Ethiopian nurses at the health center level found that approximately half (51%) of the nurses did not report an adverse drug reaction they had encountered (19). The differences in the study findings can be explained by the scope of the patient safety practices assessed and the skill level of the health science students and the different health professionals.

In this study, it was found that participants who were doing their practical clinical attachment in the surgical and obstetric units were three times more likely to self-report medical errors as compared to those practicing in other departments (AOR = 2.72, 95%CI: 1.16–6.39). This is because surgical and obstetric procedures are very complex as compared to medical and pediatric care, which involve fewer surgical techniques and procedures. It also takes considerable time for health science students to acquire competency in the surgical techniques used in surgery and obstetric departments. This finding is supported by another study that found an association between burnout and medical errors, revealing the challenging work environment in surgical wards and operating theaters (22).

In the current study, participants who had never managed a patient independently were less likely to self-report medical error (AOR = 0.24, 95%CI: 0.08–0.72). This finding can be explained by the fact that health science students who had never managed patients independently were likely to never have conducted a treatment procedure, and therefore, the chance of making medical errors will be lower. The more trainees or health professionals engage in activities, the more they are prone to make medical errors. One study among residents in the United States found that those who felt overwhelmed with work reported higher rates of suboptimal care practices (23).

This study also found the odds of reporting a medical error were lower among participants who had not ever witnessed harm to patients by colleagues or other healthcare workers (AOR = 0.12, 95%CI: 0.05–0.29) and among participants who had not ever witnessed harm harm to a close friend or family member (AOR = 0.36, 95%CI: 0.16–0.80). This is because those trainees who have encountered medical errors before are likely to be knowledgeable about the issue and will thus have higher rates of reporting any medical errors committed. On the other hand, students who had no encounter or exposure to medical errors are less likely to notice or identify medical errors when they are committed.

In general, our study has shaded some light on the status of patient safety culture among undergraduate health science students of Arba Minch University in Southern Ethiopia. Though patient safety is not a new concept, its standardization and adoption across healthcare settings is not smooth and uniform. Several other initiatives and international studies have revealed how the development of ethical and safety fields can take time and face several implementation challenges (24, 25). Our study was focused on health science students only, but including the patient safety perspectives of health professionals and patients could provide broader insights into patient safety culture adoption in the studied area.

## 5 Strengths and limitations

### 5.1 Strengths

- The study involved students from different fields of healthcare (medicine, health officer, nursing, midwifery, medical laboratory science, and anesthesia), which allows the understanding of the education curriculum across different fields.
- Standard and valid questionnaires used in other studies were adopted and adapted for this study, utilizing standards from the WHO patient safety manuals and guidelines.
- The study participants were enrolled in practical attachment in different hospitals and health centers, allowing the understanding of patient safety practice in different practical training settings.

### 5.2 Limitations

- The monocentric nature of our study, conducted solely at Arba Minch University in southern Ethiopia, could have some biases related to localization; therefore, we recommend that future research studies include multiple universities or colleges in different geographic locations that are involved in training health science students.
- The respondents might not provide accurate information pertaining to the sensitivity of medical errors.
- The cross-sectional nature of the study makes determining causality impossible.

## 6 Conclusion

A considerable number, 18.2% of participants reported having caused harm to patients while practicing. The majority of the students had good patient safety knowledge, whereas approximately two-thirds of the participants (62.5%) had a favorable attitude toward patient safety. Only just over one-third of the study participants (38.6%) had good patient safety practices.

Practical attachment units (surgery and obstetric units), having managed a patient independently, having witnessed harm to patients by colleagues or other healthcare workers, and having witnessed harm to a close friend or family member were found to be statistically significantly associated with self-reported medical errors.

## 7 Recommendations

- Quality improvement projects focusing on patient safety practice need to be conducted, and supervision and monitoring of the daily activities of undergraduate students must be done, ensuring that patient safety is assessed and documented.
- On-the-job training about patient safety and medical errors should be given to undergraduate students during practical attachment in healthcare facilities. Supervisors should actively engage in monitoring students’ practice, providing feedback, and addressing any observed errors or near misses promptly.
- Patient safety titles should be incorporated and reinforced in the curriculum for health professionals’ training.
- Hospitals and health centers where students do their practical attachment should ensure the development and implementation of patient safety documentation policies and standard guidelines and develop measures to monitor the implementation through regular clinical audits and quality improvement projects about patient safety.
- Hospitals should create an effective committee of patient safety teams involving all domains of healthcare providers including setting up ways of reporting and following up on patient safety activities.
- Academic health science training centers and healthcare facilities should consider providing psychosocial support services for students who have experienced or witnessed harm to patients, colleagues, or family members. They should also offer counseling and resources to help students cope with the emotional and psychological impact of these experiences and mitigate the risk of burnout or distress.
- Stakeholders should foster a culture of safety within healthcare education institutions, emphasizing the importance of transparency, accountability, and continuous improvement in patient care. They should encourage students to actively participate in patient safety initiatives and quality improvement projects to promote a shared commitment to safe practice.
- Training institutions should establish support systems for students to report and discuss medical errors and adverse events in a confidential and non-punitive environment. Open dialogue is recommended to reflect on experiences related to patient harm, with a focus on learning from errors to prevent recurrence.

## Data availability statement

The raw data supporting the conclusions of this article will be made available by the authors, without undue reservation.

## Ethics statement

The studies involving humans were approved by Institutional Research Ethics Review Committee of the College of Medicine and Health Sciences of Arba Minch University. The studies were conducted in accordance with the local legislation and institutional requirements. The participants provided their written informed consent to participate in this study.

## Author contributions

KT: Writing – review & editing, Writing – original draft, Supervision, Software, Methodology, Investigation, Funding acquisition, Formal analysis, Data curation, Conceptualization. FD: Writing – original draft, Supervision, Methodology, Investigation, Funding acquisition, Data curation. SA: Writing – original draft, Methodology, Funding acquisition, Data curation. TM: Writing – original draft, Methodology, Investigation, Funding acquisition, Data curation, Conceptualization. SD: Writing – original draft, Validation, Supervision, Project administration, Methodology, Investigation, Funding acquisition, Data curation, Conceptualization. WB: Writing – review & editing, Writing – original draft, Validation, Supervision, Software, Project administration, Methodology, Investigation, Funding acquisition, Formal analysis, Data curation, Conceptualization.
