# Supplementary material for: Factors associated with self-reported medical errors among undergraduate health science students in southern Ethiopia
Source: Front Med (Lausanne). 2024 Jun 13;11:1354270. doi: 10.3389/fmed.2024.1354270 (PMC11208695; doi:10.3389/fmed.2024.1354270)
Supplement: Supplementary file 1 [file Table_1.docx]

Supplementary Material

Factors associated with self-reported medical errors among undergraduate health science students in southern Ethiopia.

Kusse Koirita Toitole*, Fekade Tesfaye Danaso, Saron Assefa Alto, Tofik Mohammed, Sisay Dejene, Wanzahun Godana Boynito

*** Correspondence:** Corresponding Author: [kussekoirita@gmail.com](mailto:kussekoirita@gmail.com)

# Supplementary Figures and Tables

**Structured Questionnaire for Knowledge, Attitude, and Practice (KAP) Survey on Patient Safety**

**Part I. Knowledge questions**

| S.N. | Question | Response | |
| --- | --- | --- | --- |
| 1 | Patient safety is the prevention of errors and adverse effects to patients associated with health care | Yes | No |
| 2 | The way healthcare workers use different medical equipment can lead to errors on patients |  |  |
| 3 | The systematic approach of minimizing healthcare-related errors can allow us to examine healthcare system-related problems as a whole rather than focusing on an individual level |  |  |
| 4 | Effective teamwork in healthcare delivery can have an immediate and positive impact on patient safety |  |  |
| 5 | Learning from error can occur at both an individual level and organizational level through error reporting and analysis |  |  |
| 6 | One way for professionals to help prevent adverse events is to identify areas more likely to have errors |  |  |
| 7 | Patient safety requires the ability to measure patient outcomes and test whether the interventions used to fix a problem were effective. |  |  |
| 8 | When health professionals involve patients and caregivers in health care, less adverse events are likely to occur |  |  |
| 9 | Human sources of infectious agents include patients, healthcare workers, and visitors. |  |  |
| 10 | Encouraging patients to keep a written record of the medications that they take and details of any allergies help to reduce drug adverse events |  |  |

**Part II. Attitude Questions**

| S.N | Question | Response | | | | |
| --- | --- | --- | --- | --- | --- | --- |
|  |  | Strongly agree | Agree | Neutral | Disagree | Strongly disagree |
|  |  | 5 | 4 | 3 | 2 | 1 |
| 1 | Medical errors are nearly always caused by multiple factors |  |  |  |  |  |
| 2 | A health organization’s culture is a significant contributor to patient safety. |  |  |  |  |  |
| 3 | Effective communication is important for promoting patient safety |  |  |  |  |  |
| 4 | Appreciating the roles of different team members will help in the reduction of errors and improvement of healthcare |  |  |  |  |  |
| 5 | A systematic approach to learning from failures is the best response for ensuring patient safety |  |  |  |  |  |
| 6 | Disclosure of medical errors to patients and families is valuable to health-care quality |  |  |  |  |  |
| 7 | Human factors is about understanding human limitations and designing the workplace and the equipment we use to allow for variability among humans and their activities |  |  |  |  |  |
| 8 | The process of delivering medications to patients often involves a range of health-care professionals. |  |  |  |  |  |
| 9 | Prevention of infection must always be the priority of all health-care workers and, as such, is a key component of patient safety programs. |  |  |  |  |  |
| 10 | Understanding the multiple causes of adverse events requires the use of methods designed to identify all the likely causes. |  |  |  |  |  |

**Part IV. Practice Questions**

| S.N |  | Response | | | | |
| --- | --- | --- | --- | --- | --- | --- |
|  |  | Not competent | Somewhat competent | Competent | Proficient | Expert |
|  |  | 1 | 2 | 3 | 4 | 5 |
| 1 | I can routinely use checklists in my clinical practice when there is an evidence-based way of selecting or implementing treatment. |  |  |  |  |  |
| 2 | I can describe the role of human factors in patient safety |  |  |  |  |  |
| 3 | Using a systems approach, I can analyze the multiple factors underpinning adverse events. |  |  |  |  |  |
| 4 | If I am a team leader, I can effectively facilitate, coach, and coordinate the activities of other team members |  |  |  |  |  |
| 5 | I can respond appropriately to patients and families after an adverse event |  |  |  |  |  |
| 6 | I can appropriately inform patients and obtain informed consent for treatments and interventions, and support patients in making informed choices |  |  |  |  |  |
| 7 | I know what to do if exposed to blood or other bodily fluids |  |  |  |  |  |
| 8 | I can use standard precautions to prevent and control healthcare-associated infections (HCAI) |  |  |  |  |  |
| 9 | I can follow verification processes to avoid wrong patient, wrong side, and wrong procedure errors (e.g., a surgical checklist) |  |  |  |  |  |
| 10 | I can list some medications used in our area that are associated with high risks of adverse events. |  |  |  |  |  |
